# Renoprotective Effects of Antroquinonol in Rats with N^ω^-Nitro-l-Arginine Methyl Ester-Induced Hypertension

**DOI:** 10.3390/nu10101521

**Published:** 2018-10-17

**Authors:** Jiun-Rong Chen, Jung Ko, Wan-Ju Yeh, Wen-Chih Huang, Hsin-Yi Yang

**Affiliations:** 1 Department of Nutrition and Health Sceinces, School of Nutrition and Health Sciences, Taipei Medical University, Taipei 110, Taiwan; syunei@tmu.edu.tw (J.-R.C.); a532732002@hotmail.com (J.K.); 2 Department of Food Science, College of Agriculture, Tunghai University, Taichung 407, Taiwan; b506093091@tmu.edu.tw; 3 Department of Anatomic Pathology, Far Eastern Memorial Hospital, New Taipei City 220, Taiwan; pathology.taipei@gmail.com; 4 College of Nursing, National Taipei University of Nursing and Health Sciences, Taipei 112, Taiwan; 5 Department of Nutrition, I-Shou University, Kaohsiung 82445, Taiwan

**Keywords:** antroquinonol, hypertension, nitric oxide, Nrf-2, inflammation

## Abstract

Endothelial dysfunction leads to elevation of blood pressure and vascular remodeling, which may result in tissue injuries. The aim of this study was to investigate the mechanisms and effects of antroquinonol on hypertension and related renal injuries. Rats were fed water containing 25 mg/kg/day N^ω^-nitro-l-arginine methyl ester (L-NAME) to induce hypertension, and a diet with or without antroquinonol (20 or 40 mg/kg/day) for a 9-week experiment. During the experimental period, antroquinonol reduced the elevation of systolic and diastolic blood pressure. At the end of the study, we found that the antroquinonol groups had lower serum creatinine, renal endothelin-1, angiotensin II, and malondialdehyde levels and arteriole thickening. We found that the 40 mg/kg/day antroquinonol group had lower renal nicotinamide adenine dinucleotide phosphate (NADPH) oxidase activities, greater nuclear factor erythroid-2, and heme oxygenase-1 expressions. Moreover, we also found that antroquinonol decreased proinflammatory cytokine concentrations in the kidney by modulating the nuclear factor-κB pathway. These results suggest that antroquinonol may ameliorate hypertension and improve renal function by reducing oxidative stress and inflammation in rats with endothelial dysfunction.

## 1. Introduction

High blood pressure may result in vascular remodeling and cause tissue injuries in the renal and cardiovascular systems. Endothelial dysfunction, which can be found in the early stage of hypertension, is an important factor that elevates blood pressure through activation of inflammatory responses and increases in reactive oxidative species (ROS) [1]. Reducing nitric oxide (NO) bioavailability and increasing free radical production dysregulates the excretion of angiotensin (Ang) II and endothelin (ET)-1 [2]. Ang II, a vasoconstrictor, can activate NADPH oxidase (NOX) and generate ROS. Recent studies demonstrated that oxidative stress is also related to elevated blood pressure and may drive the progression of hypertensive complications [3]. Nuclear factor erythroid (Nrf)-2 plays a crucial role in antioxidative responses in vivo, and Nrf-2 activators may be beneficial in treating hypertension [4]. Modulating p38/Nrf-2 signaling pathways resulted in an increase in heme oxygenase (HO)-1 activity and alleviated inflammation in the endothelium [5].

*Antrodia camphorata* is a special mushroom in Taiwan that is traditionally used in Chinese medicinal cuisine or Chinese remedies to treat hepatitis and cancer due to its antioxidative and anti-inflammatory effects [6]. Antroquinonol, one of the main bioactive ubiquinone derivatives in *A. camphorata*, exhibited anti-inflammatory effects in lipopolysaccharide-stimulated RAW 267.4 cells [7] and reduced the production of tumor necrosis factor (TNF)-α and interleukin (IL)-1β in mice with nephritis [8]. Antroquinonol also reduced oxidative stress and inflammation via regulating the Nrf-2 signaling pathway [9]. However, we found no reports about its effects on blood pressure regulation. Thus, we aimed to use an NO-deficiency-induced endothelial dysfunction model to investigate the effects of antroquinonol on hypertension and related renal injuries, and to clarify possible underlying mechanisms. 

## 2. Materials and Methods 

### 2.1. Experimental Design 

Forty 8-week-age male Wistar rats were purchased from the National Laboratory Animal Breeding and Research Center (Taipei, Taiwan) and maintained in a room at 22 ± 2 °C with 55% ± 5% humidity and a 12 h light–dark cycle. The experimental protocols were reviewed by the Institutional Animal Care and Use Committees of Taipei Medical University, investigators followed protocols described in the “Guide for the Care and Use of Laboratory Animals”, and all protocols were approved by the Institutional Animal Care and Use Committee (IACUC). After 1-week of adaptation, rats were divided into four groups for a 9-week experiment. The control (C) group was fed a standard normal diet (5001-laboratory rodent diet, LabDiet, St, Louis, MO) and distilled water; the hypertensive (H) group and experimental (A & B) groups were fed water containing 25 mg/kg/day N^ω^-nitro-l-arginine methyl ester (L-NAME), and diets containing different doses of antroquinonol, which was an isolated light yellow oil, characterized as previously reported [10] (GoldenBiotech, New Taipei City, Taiwan). Antroquinonol was reported to be effective at a dose of 10–50mg/kg/day [11]. The four groups were the C group (*n* = 10, fed normal diet and ddH_2_O), the H group (*n* = 10, fed 25 mg/kg/day L-NAME), the A group (*n* = 10, fed 25 mg/kg/day L-NAME + 20 mg/kg/day antroquinonol), and the B group (*n* = 10, 25 mg/kg/day L-NAME + 40 mg/kg/day antroquinonol). Food and water intake were recorded daily, and body weights were recorded weekly during the experimental period. At the end of the study, all rats were sacrificed, and blood and kidney tissue samples were collected for analysis.

### 2.2. Blood Pressure Measurement 

We measured the rats’ blood pressure at 0, 3, 5, 7, and 9 weeks using a non-invasive tail-cuff method (MK-2000ST; Muromachi Kikai, Tokyo, Japan). After being starved for 8 h, at least three readings were collected to calculate the mean systolic and diastolic blood pressures.

### 2.3. Blood Collection and Analysis 

Serum samples were collected after the centrifugation of blood from the interior vena cava and stored at –80 °C before being analyzed. Serum alanine aminotransferase (ALT), aspartate aminotransferase (AST), albumin, creatinine, and blood urea nitrogen were determined using a Roche Modular P800 (Roche Diagnostics, Indianapolis, IN, USA). Plasminogen activator inhibitor (PAI)-1, Ang II, and ET-1 were analyzed with enzyme-linked immunosorbent assay (ELISA) kits (RK003A, HYPHEN BioMed, Neuville-sur-Oise, France; EKE-002-12, Phoenix Pharmaceuticals, Burlingame, CA, USA; DET100, R&D Systems, Minneapolis, MN, USA).

### 2.4. Renal Tissue Collection and Analysis

To analyze renal malondialdehyde (MDA), ET-1, TNF-α, and IL-1β concentrations, kidney samples were homogenized in a 400 mM phosphate buffer (pH 7.2) containing 340 mM sucrose, 900 mM NaCl, and protease inhibitors. Lipid peroxidation was evaluated by the thiobarbituric acid-reactive substance method with spectrophotometric detection at 532 nm, and results are expressed as MDA equivalents [12]. ET-1, TNF-α, and IL-1β were analyzed using enzyme-linked immunosorbent assay (ELISA) kits (DET100, DY501, & 510, R&D Systems). 

To analyze glutathione levels and the antioxidative capacity, kidney samples were homogenized in phosphate-buffered saline (PBS) containing 1 mM ethylenediaminetetraacetic acid (EDTA) (pH 7.4) and centrifuged at 10^4^× *g*, at 4 °C for 15 min. The supernatant was collected for measurement of the total glutathione and ferric-reducing antioxidant power (FRAP) assays (703002, Cayman Chemical, Ann Arbor, MI, USA; STA-859, CELL BIOLABS Inc., San Diego, CA, USA).

To measure NADPH oxidase (NOX) activity, kidney samples were homogenized in an extraction solution containing 0.25 M sucrose, 50 mM hydroxyethylpiperazine ethane sulfonic acid (HEPES), 3 mM EDTA, 1 mM dithiothreitol (DTT), 3.6 mM L-cysteine, and 0.1 mM MgCl_2_, and centrifuged at 10^4^× *g*, at 4 °C for 45 min, after which, the supernatant was centrifuged at 2.03 × 10^5^× *g*, at 4 °C for 1 h. The precipitate was then resuspended with 10 mM Tris-HCL (pH 7.4), and NOX activity was detected following a previously reported method at 492 nm [13].

To analyze the protein expressions of NOX4, nuclear factor (NF)-κB, and the inhibitor of NF-κB (IκB), we homogenized kidney samples in a radioimmunoprecipitation assay (RIPA) buffer containing protease inhibitors and centrifuged them at 10^4^× *g*, at 4 °C for 10 min. Supernatants with 30 μg of protein were separated on a sodium dodecylsulfate (SDS)-polyacrylamide gel and transferred onto a polyvinylidene difluoride membrane. Nuclear and cytosol fractions were extracted using commercial kits (BioVision, Milpitas, CA, USA) for analysis of Nrf2 and HO-1 protein expressions by Western blotting. Nonspecific binding sites were blocked by incubation of the membranes overnight at 4 °C in a blocking buffer (Visual Protein, Taipei, Taiwan). After washing with PBS/Tween-20, membranes were incubated with either an anti-NOX4 antibody (ab133303, Abcam, Cambridge, UK), anti-NFκB p65 (622601, BioLegend, San Diego, CA, USA), phospho-NF-κB p65 (Ser536)(3033, Cell Signaling, Beverly, MA, USA), anti-IκB alpha antibody (ab32518, Abcam), NFE2L2 polyclonal antibody (Proteintech, Rosemont, IL, USA) or anti-HO-1 antibody (ab13248, Abcam) at room temperature for 2 h, followed by incubation with a horseradish peroxidase (HRP)-conjugated secondary antibody (Jackson ImmunoResearch, West Grove, PA, USA). Membranes were then washed and treated with a chemiluminescence detection system (ECL, PerkinElmer, Waltham, MA, USA) to develop the immune complex. The BioSpectrum AC Image systems UVP Visionwork LS Software and Image-Pro Plus 4.5 (Media Cybernetic, Bethesda, MD, USA) were used to quantify the bands. Equal loading of the total protein was verified using a commercially available monoclonal antibody against β-actin (622102, BioLegend), and the results are expressed as the ratio of protein to β-actin.

Kidney samples used for the pathological analysis were fixed in 10% formaldehyde and stained with hematoxylin and eosin (H & E). Morphological changes of renal arterioles were examined on a blinded basis by a pathologist.

### 2.5. Statistical Analysis 

Results are expressed as the mean and standard error of the mean (SEM) and were analyzed with the SAS program (vers. 9.3; Cary, NC, USA). One-way analysis of variance (ANOVA) and Fisher’s least significant difference were used to analyze data among groups at the end of the study. Changes in blood pressure during the experimental period were analyzed by a repeated-measures analysis of variance (ANOVA), and Duncan’s multiple-range test. A *p* value of <0.05 was taken as the level of statistical significance.

## 3. Results

### 3.1. Body Weight, Food Intake, and Blood Pressure 

**Figure 1:**
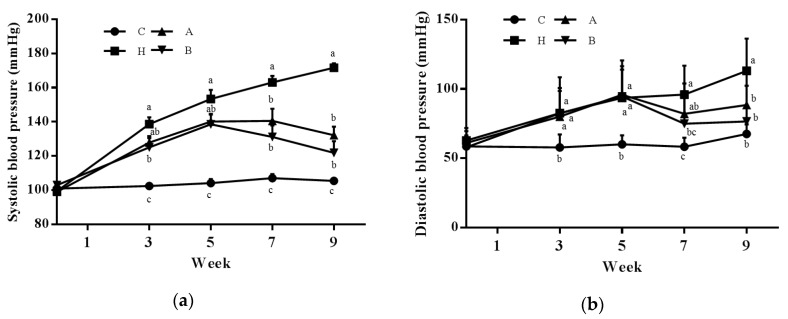
Changes in (**a**) systolic blood pressure (SBP) and (**b**) diastolic blood pressure (DBP) in rats during the experimental period. Values are presented as the mean ± standard error of the mean (SEM) (*n* = 10). ^a b c^ Values with different superscript letters at the same time point significantly differ (*p* < 0.05). C, control group (filled circles); H, hypertensive group (filled squares); A, 20 mg/kg/day antroquinonol group (filled triangles); and B, 40 mg/kg/day antroquinonol group (filled inverted triangles).

During the experimental period, we found no difference in either food or water intake among groups, and average antroquinonol intake levels were 19.9 ± 0.4 and 40.7 ± 0.4 mg/kg/day in groups A and B. No difference in initial or final body weights was found among groups. Group H had significantly higher systolic blood pressure (SBP) and diastolic blood pressure (DBP) than group C. Group B showed significantly lower SBP than group H from week 3, and both antroquinonol groups had significant lower SBP and DBP values than group H at the end of the study (Figure 1).

### 3.2. Endothelial Dysfunction and Kidney Injury

Chronic L-NAME treatment significantly elevated serum creatinine, PAI-1, ET-1, and MDA levels, while the antroquinonol-consuming A and B groups had lower serum creatinine and Ang II levels, lower kidney ET-1 levels, and higher ferric reducing antioxidative capacities than group H. In addition, group B also had lower ALT and PAI concentrations than group H (Table 1). 

**Figure 2:**
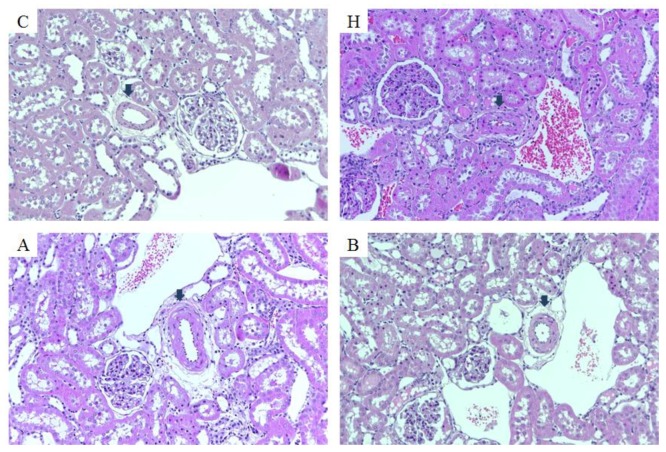
Pathohistological changes in the kidney of rats at the end of the study. C, control group; H, hypertensive group; A, 20 mg/kg/day antroquinonol group; and B, 40 mg/kg/day antroquinonol group. Group C showed normal intrarenal arterioles. Group H had prominent intimal thickening with a reduced vascular lumen, while group A had mild to moderate thickening of the arterioles, and group B (25 mg/kg/day of L-NAME with 40 mg/kg/day antroquinonol in the diet) showed mild thickening of the arterioles. (H & E stain, original magnification × 200).

In the histopathological analysis, we found that a chronic NO deficit led to intimal thickening with reduced vascular lumen present in renal arterioles, and antroquinonol alleviated arteriole thickening (Figure 2).

### 3.3. Oxidative Stress 

**Figure 3:**
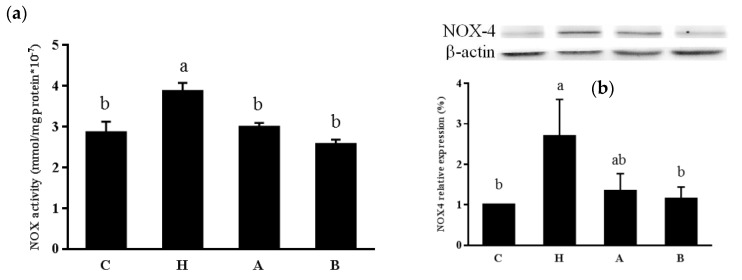
Kidney (**a**) NADPH oxidase (NOX) activity and (**b**) NOX4 protein expression in rats. Values are presented as the mean ± SEM (*n* = 10). ^a b c^ Values with different superscript letters at the end of the study significantly differ (*p* < 0.05). C, control group; H, hypertensive group; A, 20 mg/kg/day antroquinonol group; and B, 40 mg/kg/day antroquinonol group.

**Figure 4:**
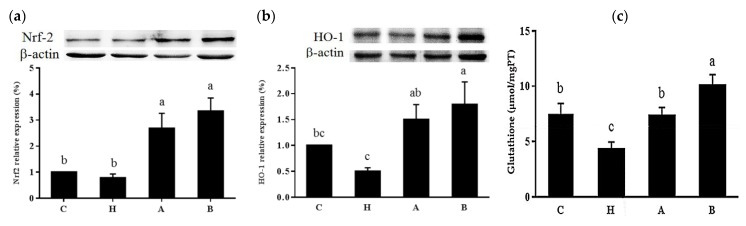
Kidney (**a**) nuclear factor erythroid 2-related factor-2 (Nrf-2), (**b**) cytosolic heme oxygenase (HO)-1 protein expressions, and (**c**) total glutathione (GSH) level in rats. Values are presented as the mean ± SEM (*n* = 10). ^a b c^ Values with different superscript letters at the end of the study significantly differ (*p* < 0.05). C, control group; H, hypertensive group; A, 20 mg/kg/day antroquinonol group; B, 40 mg/kg/day antroquinonol group.

L-NAME significantly increased kidney NOX activity and NOX4 protein expressions, while both antroquinonol groups exhibited no differences in NOX activity or NOX4 protein expression compared to group C (Figure 3). We also found that antroquinonol increased kidney nuclear Nrf-2 and cytosolic HO-1 expressions, increased glutathione concentration and FRAP, and decreased MDA levels in the kidney (Figure 4, Table 1).

### 3.4. Inflammatory Effects 

**Figure 5:**
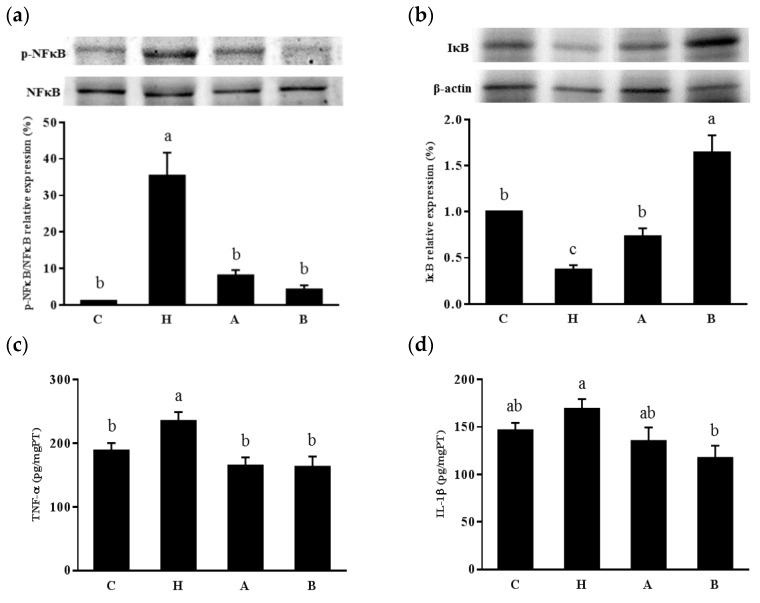
Kidney (**a**) phospho-nuclear factor (NF)-κB and NF-κB expressions; (**b**) inhibitor of NF-κB expressions (IκB), and proinflammatory cytokine; (**c**) tumor necrosis factor (TNF)-α; and (**d**) interleukin (IL)-1β levels in rats. Values are presented as the mean ± SEM (*n* = 10). ^a b c^ Values with different superscript letters at the end of the study significantly differ (*p* < 0.05). C, control group; H, hypertensive group; A, 20 mg/kg/day antroquinonol group; and B, 40 mg/kg/day antroquinonol group.

The L-NAME group had significantly higher renal pNFκB/NFκB and TNF-α concentration than the control group, and both antroquinonol groups had lower renal pNFκB/NFκB and TNF-α levels than group H (Figure 5).

## 4. Discussion

NO is involved in various physiological functions in the cardiovascular system, and chronic blockage of NO production will cause endothelial dysfunction, imbalance of the renin-angiotensin system, and cardiovascular remodeling, lead to systemic hypertension in experimental models, which is similar to hypertension observed in humans [14]. L-NAME is a widely used NO synthase inhibitor, and chronic treatment with L-NAME is known to decrease NO bioavailability and imbalance the excretion of other vascular tone regulators, such as Ang II and ET-1, leading to hypertension [15]. In the present study, daily treatment with 25 mg/kg L-NAME significantly elevated blood pressure from the 3rd week and also led to higher ET-1 and Ang II concentrations compared to normal rats. We found that the antroquinonol groups had lower blood pressure, and a daily intake of 40 mg/kg antroquinonol produced significantly lower circulating ET-1 and Ang II levels. Elevated blood pressure and increased oxidative stress may further cause vascular remodeling and tissue damage [16]. After 9 weeks of L-NAME treatment in our study, rats had elevated serum creatinine and renal arteriole thickening, indicating early-stage kidney injury. We also found higher circulating PAI-1 and MDA, higher renal ET-1 and MDA levels, and a lower antioxidative capacity in group H as well. On the other hand, antroquinonol groups had lower creatinine and kidney ET-1 and MDA concentrations, and showed a greater antioxidative capacity. These results suggest that antroquinonol may ameliorate L-NAME-induced hypertension and kidney tissue injuries.

Oxidative stress and proinflammatory factors play important roles in the pathogenesis and progression of hypertension. NOX is an enzyme related to ROS formation in vascular systems, and activation of NOX is associated with cardiovascular and renal remodeling [17]. Among NOX isoforms, NOX4 is mainly expressed in the kidneys and is an important source of renal oxidative stress [18]. Studies demonstrated that elevation of Ang II may increase the formation of ROS and lead to renal injuries via activating NOX4 [19]. In addition, ET-1 may also increase vascular ROS levels, which causes endothelial dysfunction and hypertension, through the activating NOX [20]. In our study, we found that the L-NAME group had higher renal NOX4 protein expression and activity, while no difference in NOX4 activity from the antroquinonol groups was found compared to the control group. Consistently, the L-NAME group also had the lowest total antoxidative capacity and highest ET-1 levels among all groups. Therefore, antroquinonol may regulate the excretion of ET-1 and Ang II, and decrease renal NOX4 expression. 

The Nrf-2 signaling pathway is also important in cellular defense against oxidative stress through regulating antioxidants, such as HO-1, glutathione, and so on [21]. Modulation of Nrf-2 expression may help to normalize the antioxidative status and decrease renal injury and inflammation in hypertensive animal models [22]. HO-1 is a cytoprotective enzyme that generates biliverdin, ferrous iron, and carbon dioxide, and exhibits antioxidative anti-inflammatory effects [23]. In rats with L-NAME-induced hypertension, enhancement of HO-1 activity ameliorated renal dysfunction by suppressing oxidative and inflammatory mediators [24]. Glutathione is one of the main tissue protective antioxidants in vivo, and depletion of glutathione leads to increased blood pressure and affects normal renal function [25]. A previous study also found that L-NAME treatment decreased renal glutathione and increased serum creatinine levels [26]. Antroquinonol was reported to have the potential to activate Nrf-2, decrease ethanol-induced oxidative stress in hepatic cells [27], and ameliorate the progression of focal segmental glomerulosclerosis in mice through reducing oxidative stress [9]. However, no previous study has reported the effects of antroquinonol on blood pressure management. In our study, we found that antroquinonol upregulated renal Nrf-2 expression and also increased HO-1 expression and glutathione concentrations. These results suggest that antroquinonol may also improve hypertension through modulating Nrf-2 signaling and related antioxidative responses.

Activation of the inflammatory response is thought to be one of the factors that drives the progression of hypertension. In spontaneously hypertensive rats, activation of renal NF-κB and interstitial inflammation are found during the prehypertensive stage [28]. ROS can activate the NF-κB pathway by stimulating the isolation of NF-κB from its inhibitory protein, IκB, and its translocation into nuclei, resulting in the activation and excretion of proinflammatory cytokines and oxidative species [29]. The generation of free radicals, cytokines, and Ang II further stimulates the activation of the NFκB pathway, and this vicious cycle may lead to the progression of cardiorenal remodeling and tissue injuries [30,31]. A previous study demonstrated that antroquinonol reduced NF-κB protein expression in rat gliomas [32] and ameliorated renal damage by inhibiting NF-κB and downstream cytokine excretion [9]. In the present study, we found that antroquinonol modulated renal expressions of NF-κB and IκB, and the 40-mg/kg/day antroquinonol group had significantly lower renal TNF-α and IL-1β concentrations than the L-NAME group. Therefore, antroquinonol may also retard the progression of hypertension and renal dysfunction by regulating the NF-κB signaling pathway. Antroquinonol has been studied for its anticancer effects with very mild adverse events reported [33]. In this study, we first reported the effects of antroquinonol in an endothelial dysfunction rodent model and found no adverse effects at the dosage of 40-mg/kg/day in rats. However, there were also some reports of the effects of antroquinonol on blood sugar regulation and inflammation-related diseases [9,11,34], although studies and evidence in vivo remain scarce. Further studies are needed to clarify the effects of antroquinonol on metabolic disorders, and the results may be used in the treatment of chronic diseases.

## 5. Conclusions

**Figure 6:**
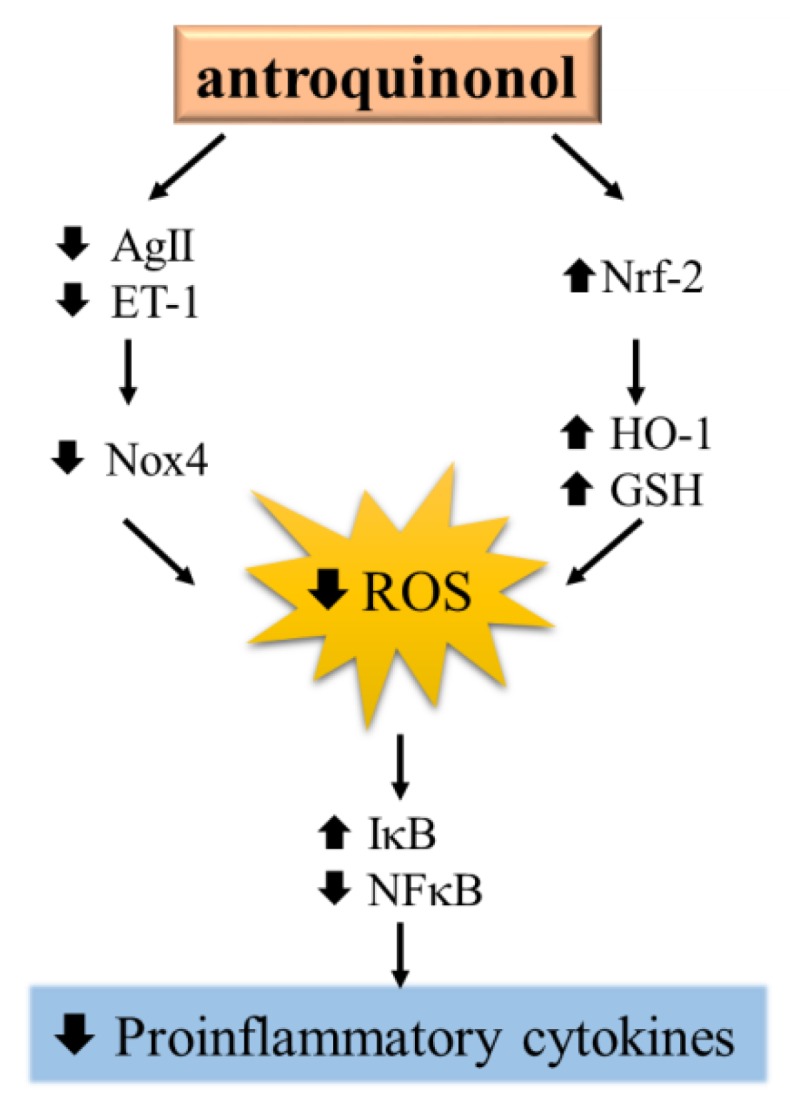
Antroquinonol improved oxidative stress and inflammation by upregulating the nuclear factor erythroid 2-related factor 2 (Nrf)-2 pathway and modulating NADPH oxidase-4 (NOX4) activity in rats with endothelial dysfunction.

Taken together as shown in Figure 6, our results revealed that antroquinonol can ameliorate hypertension and improve renal function by reducing oxidative stress and inflammation by upregulating the Nrf-2 pathway and modulating NOX4 activity in rats with endothelial dysfunction.

## Figures and Tables

**Table 1:** Serum and kidney tissue injury, and oxidative and hypertensive biomarkers.

	C	H	A	B
Serum				
ALB (g/L)	37.40 ± 0.56	36.70 ± 0.60	36.20 ± 1.02	36.40 ± 0.65
BUN (mg/dL)	14.60 ± 0.50	15.80 ± 0.79	15.50 ± 0.52	15.20 ± 0.59
Cr (mg/dL)	0.29 ± 0.02 ^b^	0.38 ± 0.04 ^a^	0.29 ± 0.02 ^b^	0.29 ± 0.02 ^b^
AST (IU/L)	73.00 ± 2.10	81.80 ± 4.42	77.50 ± 4.12	74.80 ± 3.59
ALT (IU/L)	34.80 ± 1.60 ^b^	50.50 ± 4.17 ^a^	46.60 ± 2.85 ^a^	35.30 ± 2.77 ^b^
PAI-1 (ng/ml)	0.35 ± 0.03 ^c^	0.66 ± 0.09 ^a^	0.53 ± 0.04 ^ab^	0.44 ± 0.07 ^bc^
ET-1 (pg/mL)	0.95 ± 0.06 ^b^	1.19 ± 0.10 ^a^	0.97 ± 0.10 ^ab^	0.89 ± 0.05 ^b^
AngII (pg/mL)	358.3 ± 29.9 ^ab^	402.7 ± 65.2 ^a^	332.2 ± 31.6 ^b^	310.9 ± 22.6 ^b^
MDA (μmol/L)	11.76 ± 0.60 ^b^	16.05 ± 1.81 ^a^	12.71 ± 0.57 ^b^	12.05 ± 0.50 ^b^
Kidneys				
ET-1 (pg/mg protein)	1.2 ± 0.1 ^b^	1.4 ± 0.1 ^a^	1.0 ± 0.1 ^bc^	0.9 ± 0.1 ^c^
MDA (nmol/mg protein)	2.3 ± 0.1 ^ab^	2.5 ± 0.1 ^a^	2.1 ± 0.1 ^bc^	1.8 ± 0.1 ^c^
FRAP (nmol/mg protein)	59.0 ± 4.0 ^b^	38.7 ± 3.8 ^c^	59.1 ± 4.2 ^b^	95.4 ± 10.4 ^a^

Values are presented as the mean ± SEM (*n* = 10). ^a b c^ Values with different superscript letters at the end of the study significantly differ among groups (*p* < 0.05). C, control group; H, hypertensive group; A, 20 mg/kg/day antroquinonol group; B, 40 mg/kg/day antroquinonol group. ALB, albumin; BUN, blood urea nitrogen; Cr, creatinine; AST, aspartate aminotransferase; ALT, alanine aminotransferase; PAI-1, plasminogen activator inhibitor-1, ET-1, endothelin-1; Ang II, angiotensin II; MDA, malondialdehyde; and FRAP, ferric-reducing antioxidant power.

## Abbreviations

NO	nitric oxide
Ang	angiotensin
ET	endothelium
NOX	NADPH oxidase
Nrf	nuclear factor erythroid
HO	heme oxygenase
TNF	tumor necrosis factor
IL	interleukin
ALT	alanine aminotransferase;
AST	aspartate aminotransferase
PAI	plasminogen activator inhibitor
MDA	malondialdehyde
NF-κB	nuclear factor-κB
IκB	inhibitor of NF-κB
